# Who is afraid of ticks and tick-borne diseases? Results from a cross-sectional survey in Scandinavia

**DOI:** 10.1186/s12889-019-7977-5

**Published:** 2019-12-11

**Authors:** Daniel Slunge, Solveig Jore, Karen Angeliki Krogfelt, Martin Tugwell Jepsen, Anders Boman

**Affiliations:** 1 Gothenburg Centre for Sustainable Development, GMV, University of Gothenburg, Box 170, 40530 Gothenburg, Sweden; 2 Norwegian Institute of Public Health, Oslo, Norway; 3 Statens Serum Institut, Copenhagen, Denmark; 4 Department of Science and Environment, Roskilde University, Roskilde, Denmark; 5 Statens Serum Institut, Copenhagen, Denmark; 6 Department of Economics, University of Gothenburg, Gothenburg, Sweden

**Keywords:** Risk perception, Knowledge, Tick, Lyme borreliosis, Tick-borne encephalitis (TBE), Ixodes ricinus

## Abstract

**Background:**

In Scandinavia, the distribution of ticks is expanding and tick-borne diseases constitute growing health risks. While the probability of getting a tick-borne disease after a tick bite is low, the health impacts can be large. This, as well as other characteristics of these diseases make tick-related risks difficult for laypeople to assess and perceived risk may differ substantially from actual risk. Understanding risk perceptions is important since it is the perceived risk, rather than actual risk, that determine behaviour and even more so for new and emerging risks. The aim of this study is to investigate knowledge and risk perceptions related to tick bites and the tick-borne diseases Lyme borreliosis (LB) and tick-borne encephalitis (TBE). By analysing risk perceptions and knowledge, the study helps inform the development of public health strategies in response to the increasing incidence of these diseases in Scandinavia.

**Methods:**

Two thousand, six hundred sixty-eight respondents in Denmark, Norway and Sweden answered an online questionnaire with 48 questions, including 7 questions on risk perceptions and 9 knowledge questions. Chi-squared tests were used to analyse statistical differences between country sub-samples, gender and age groups. A multivariate regression model was used to analyse factors associated with risk perceptions.

**Results:**

Risk perceptions were on average high in comparison with scientific estimates, with respondents grossly overrating the probability of contracting LB or TBE if bitten by a tick. Also, the average perceived seriousness of a single tick bite and of getting LB or TBE was high. Knowledge on the other hand was low, especially among men and the youngest age group (18–29 years). Higher levels of knowledge about tick-borne diseases were associated with lower perceived seriousness of tick bites and LB and higher perceived seriousness of TBE. Also, having been diagnosed with LB was negatively associated with the perceived seriousness of LB.

**Conclusions:**

Our results indicate that informing about ticks and tick-borne diseases would be a relevant public health strategy as it could make risk perceptions better aligned with actual risk.

Should the TBE virus spread further in Denmark and Norway, increasing knowledge about TBE vaccination would be especially important.

## Background

Understanding how risk perceptions and knowledge develop in response to emerging or changing health risks is important in the development of public health strategies [1–3]. This may be particularly true for risks that are new, involuntary, difficult to control or potentially catastrophic, or that evoke strong feelings at the moment of decision-making. For these types of hazards, laypeople’s risk perceptions are often high relative to expert assessments [4–6]. Media, personal networks and other social mechanisms may amplify the perceived risks, further widening the gap between the risk perceptions of laypeople and experts [7].

The spread of tick-borne infectious diseases constitute rapidly growing health risks in Europe and elsewhere [8, 9]. Lyme borreliosis (LB) infection is caused by the spirochete bacteria of the *Borrelia burgdorferi* sensu lato (s.l) complex. While symptoms can be mild or absent for some individuals, they can be severe for others (including arthritis, cardiac and neurological problems), especially if not treated early. There is no vaccine available on the market, but LB infections can be treated with antibiotics [8]. Tick-borne encephalitis (TBE) is caused by a flavivirus transmitted to humans by ticks and the virus may cause severe infection of the central nervous system. 27–40% of those diagnosed with TBE suffer serious, long-term cognitive and neuropsychiatric impairments [10, 11]. There is no treatment once infected, but the disease is preventable as effective vaccines are available [9].

Risks related to tick exposure and tick-borne diseases display several of the properties that make them difficult for laypeople to assess. They are relatively new to many people, they have potentially serious health impacts, the probability of getting LB or TBE after a tick bite is very low, and the risk is difficult to control. Thus, it is not surprising that recent studies indicate that laypeople have higher risk perceptions than experts concerning ticks and tick-borne diseases [12, 13]. Previous literature also indicate that the perceived risk of tick bites and/or tick-borne diseases – in terms of both seriousness and probability – as well as the perceived efficacy of protective measures can have a stronger influence than actual exposure on protective behaviour [14–20]. Accordingly, studying the level of knowledge and perceptions of risk related to ticks and tick-borne diseases among the population is important for informing public health policy [21, 22].

The Scandinavian countries Denmark, Norway and Sweden provide for an interesting case study about tick-related risk perceptions as the distribution of *Ixodes ricinus*, the most common tick in Scandinavia, has expanded northwards and upwards in elevation in recent decades [23, 24]. Alongside this development, the incidence of LB and TBE has increased [25, 26]. There is a notable heterogeneity in TBE disease incidence in Scandinavia, with the highest incidence found in Sweden, especially in some parts of Sweden including the capital region around Stockholm.

The aim of the present study is to investigate knowledge and factors associated with risk perceptions related to tick bites, LB and TBE in Denmark, Norway and Sweden. By analysing how risk perceptions and knowledge vary between countries, age and gender, the study also aims at informing the development of public health strategies in response to the growing disease incidence.

Many of the previous studies on tick related risk perception were conducted by surveying relatively small samples in endemic areas [12, 20, 27–30] or among respondents with common backgrounds or recreational interests [19]. In contrast, this study uses a sample of a total of 2668 respondents, representative of the populations of Denmark, Norway and Sweden. To our knowledge, this is the largest sample used for studying knowledge and risk perceptions related to ticks, TBE and LB so far. This allows for a more comprehensive analysis of risk perceptions and knowledge in the population as a whole. As there is currently very limited scientific evidence on the level of knowledge and risk perceptions related to ticks and tick-borne disease in the Scandinavian population, the study can inform the development of public health strategies.

## Methods

### Study design

As part of a larger Scandinavian research project on ticks and tick-borne diseases (Scandtick Innovation), a web-based survey was designed to study exposure to and experience of ticks and tick bites, protective behaviour and risk perceptions among people in Denmark, Norway and Sweden.^1^ The survey instrument included 48 questions and was informed by previous efforts to study the issues in focus [13–15, 18–20, 31]. It included questions in the following categories: exposure to ticks; having had a tick-borne disease; knowledge on tick-related issues, general trust and risk preferences; protective behaviour related to tick bites; recreational behaviour; and demographic characteristics (age, gender, education, income and country of residence). The present article will focus particularly on the subset of the questions in the survey relating to risk perceptions and tick-related knowledge.

Risk perceptions were elicited using seven questions where respondents were asked to indicate their perceived risk on an ordinal scale. Such scales have been found to be good instruments for measuring probabilistic expectations and risk perceptions [32, 33]. We divided risk perceptions into two parts: the probability of an event occurring and the severity of the event if it were to occur. Respondents were asked to state the perceived probability of getting a tick bite in the next 12 months and the probability of contracting LB or TBE if bitten by a tick, using a scale from 0 to 100. The perceived severity of getting a tick bite, contracting LB and contracting TBE was elicited through three separate questions where answers were reported on a 0–10 scale. Respondents were also asked ‘How willing are you to take risks, in general?’ (scale 0–10) as this general risk question has previously been found to be a good predictor of actual risk-taking behaviour [32].

Nine questions in the survey concerned the level of knowledge about ticks, LB and TBE. As people’s ability to recognise a tick is important for protective practices, similar to an earlier study [19] we showed respondents five pictures, three of which showed ticks in different life stages and two showed insects, and asked respondents to identify which pictures showed a tick.

Respondents who reported having heard of LB or TBE were asked three questions to test their knowledge about LB and three questions to test their knowledge about TBE. The knowledge questions are further described in Section 3.

### Data collection

Taking budget restrictions into account, our aim was to recruit a sample that would be representative in terms of age (18–29, 30–44, 45–59 and above 60), gender and region of residence and that would consist of around 750 respondents from Denmark, 750 from Norway and 1000 from Sweden (the higher desired number of respondents in Sweden reflects the country’s larger population). The targets for each sub-quota within each country were based on the demography of the respective country, extracted from the national registries [34–36]. For each sub-quota the respondents were randomly selected from national telephone registries. 5096 persons in Denmark, 7194 in Norway and 9901 in Sweden were initially telephoned and asked about willingness to participate in the survey.

Of those contacted, 1436 (28%), 1518 (21%) and 2037 (21%) were reached and willing to participate, in Norway, Denmark and Sweden respectively. In October 2016, the web-based survey^2^ was sent to these respondents, simultaneously in all three countries. 157 (11%), 250 (16%) and 214 (11%) of the emails bounced back, which means that 1279, 1268 and 1823 people actually received the email in the three studied countries. Of these, a total of 2668 completed the survey (783, 789, and 1096), corresponding to a response rates of 61, 62 and 60% of those who received the email, respectively. Statens Serum institute in Denmark, the Norwegian Public Health Institute and the University of Gothenburg in Sweden administered the survey in the respective countries. The data collection was facilitated by the Epinion survey company.

### Data analysis

We analyse statistical differences between the country sub-samples for all variables included in the analysis. For variables regarding risk perceptions and knowledge, we also analyse statistical differences related to gender and age group. We use Chi-squared tests for dummy variables and t-tests for continuous variables to analyse statistically significant differences. We analyse factors associated with the perceived seriousness of getting a tick bite, LB or TBE through three separate regression models. While the dependent variables are ordinal in character (0–10 scale) we use a linear regression model rather than an ordinal regression model for ease of interpretation. Using an ordinal logit model yields very similar results as the Ordinary Least Square model in terms of signs and levels of statistical significance of the associated explanatory variables.

We control for potential multicollinearity among the explanatory variables by using the variance inflation factor (VIF) test. Since our variable relating to correct identification of tick pictures had VIF values greater than 10 – a commonly used benchmark in statistical analysis – this variable was dropped from the multivairate analysis. The sample size in the multivariate regression analyses were 2581, 2488 and 1769 respectively, depending on the number of respondents who had responded to all the questions (variables) included in the regression model. For example, in the analysis of the perceived seriousness of getting TBE, we excluded 811 respondents who had never heard of the disease or ‘did not know’ if they had heard of it. The statistical analysis was performed using STATA version 15.

## Results

### Descriptive statistics

Descriptive statistics as well as tests for statistical differences between the country subsamples are reported in Table 1.

**Table 1:** Descriptive statistics (mean values)

VARIABLES	[1]	[2]	[3]	[4]	[5]	[6]	[7]	[8]	*P*-values^b^	*P*-values^b^	*P*-values^b^
	Denmark	Norway	Sweden	Scandinavia	sd^a^	min	max	N^c^	H_0_:μ_1_ = μ_2_	H_0_:μ_1_ = μ_3_	H_0_:μ_2_ = μ_3_
Age of respondent	50.49	47.16	48.18	48.56	16.29	18	99	2668	0.000	**0.007**	**0.537**
Female respondent	0.536	0.515	0.526	0.526	0.499	0	1	2668	0.386	**0.670**	**0.610**
Has attended university	0.772	0.702	0.616	0.687	0.464	0	1	2616	0.002	**0.000**	**0.000**
General willingness to take risks	4.876	4.949	4.558	4.767	2.307	0	10	2668	1.000	**0.010**	**0.001**
Likelihood of being bitten by tick in next 12 months	26.49	28.71	38.64	32.13	31.85	0	100	2660	0.487	**0.000**	**0.000**
Likelihood of contracting LB if bitten by tick	16.09	18.54	20.04	18.44	19.51	0	100	2529	**0.048**	**0.000**	0.328
Likelihood of contracting TBE if bitten by tick	10.38	14.80	12.72	13.00	17.48	0	100	1822	**0.002**	**0.001**	1.000
Perceived seriousness of one tick bite	5.844	5.225	5.277	5.429	2.515	0	10	2632	**0.000**	**0.000**	1.000
Perceived seriousness of contracting LB	8.561	8.127	7.193	7.863	2.061	0	10	2538	**0.000**	**0.000**	**0.000**
Perceived seriousness of contracting TBE	9.243	8.677	9.018	9.032	1.574	0	10	1801	**0.055**	**0.020**	1.000
Ever heard of Lyme borreliosis	0.974	0.925	0.984	0.964	0.186	0	1	2668	**0.000**	0.123	**0.000**
Ever heard of TBE	0.640	0.373	0.969	0.696	0.460	0	1	2668	**0.000**	**0.000**	**0.000**
Number of correctly identified tick pictures	3.504	3.641	3.952	3.729	1.114	0	5	2668	**0.041**	**0.000**	**0.000**
Correctly identified picture 1 (adult female tick)	0.444	0.499	0.562	0.509	0.500	0	1	2668	**0.029**	**0.000**	**0.007**
Correctly identified picture 2 (insect)	0.782	0.861	0.911	0.858	0.349	0	1	2668	0.198	**0.000**	**0.001**
Correctly identified picture 3 (tick nymph)	0.497	0.445	0.584	0.517	0.500	0	1	2668	**0.000**	**0.000**	**0.001**
Correctly identified picture 4 (blood fed tick)	0.885	0.905	0.946	0.916	0.277	0	1	2668	**0.039**	**0.000**	**0.000**
Correctly identified picture 5 (insect)	0.897	0.932	0.949	0.928	0.258	0	1	2668	**0.013**	**0.000**	0.113
Number of correct answers to knowledge questions	2.964	2.412	4.066	3.253	1.642	0	6	2668	**0.000**	**0.000**	**0.000**
Correct answer to ‘Borrelia is contagious’	0.867	0.798	0.851	0.840	0.366	0	1	2668	**0.000**	0.330	**0.003**
Correct answer to ‘There is a vaccine against LB’	0.590	0.470	0.486	0.512	0.500	0	1	2668	**0.000**	**0.000**	0.490
Correct answer to ‘LB is treatable with antibiotics’	0.807	0.638	0.766	0.740	0.439	0	1	2668	**0.000**	**0.031**	**0.000**
Correct answer to ‘TBE is contagious’	0.521	0.246	0.850	0.575	0.494	0	1	2668	**0.000**	**0.000**	**0.000**
Correct answer to ‘There is a vaccine against TBE’	0.114	0.165	0.770	0.398	0.490	0	1	2668	**0.003**	**0.000**	**0.000**
Correct answer to ‘TBE is treatable with antibiotics’	0.065	0.095	0.342	0.188	0.391	0	1	2668	**0.029**	**0.000**	**0.000**
Has searched for information about ticks or related diseases	0.548	0.559	0.608	0.578	0.494	0	1	2668	**0.660**	**0.010**	**0.034**

Notes: ^a^sd = The standard deviation for all respondents in Scandinavia (column 4)

^b^From Chi-squared test for dummy variables and an unpaired t-test for continuous variables. P-values < 0.10 are highlighted.

^c^The number of respondents in Scandinavia, column 4

#### Socioeconomic variables

The total sample consists of a higher share of women compared with the population, but the gender distribution is quite similar across the country subsamples (see Table 1). Educational attainment, measured as the share of respondents having attended university, varies from 77% in Denmark to 70% in Norway and 62% in Sweden. In all national subsamples, we find that this share is substantially higher than in the respective general populations [34–36]. The relationship between sample and population age differs between countries. Danish respondents are on average older than the population [34]. The Swedish subsample, on the other hand, is younger than the population [36], whereas the age of the Norwegian subgroup is not significantly different from Norwegian population [35] (see Table 1). In the last section of this paper, we discuss the potential implications these differences in demographic characteristics between our sample and the population may have for the interpretation of our results.

#### Risk perceptions

There are small but statistically significant cross-country differences in the general willingness to take risks as well as in specific tick-related risks (see Table 1). A mean score of 4.6 on the general risk question among respondents in Sweden indicates that they are less willing to take risks in general than respondents in Denmark and Norway, who both had a mean score of about 4.9.

The first of the six risk perception questions related to ticks and tick-borne diseases concerned the perceived probability of getting a tick bite in the next 12 months. We find large variations across countries, with respondents in Denmark and Norway assigning the lowest value to this probability (the averages were 26 and 29 on a 100 point scale, respectively) and respondents in Sweden assigning the highest value (average 39).

Next, we asked respondents about their perceived probability of getting LB if they were bitten by a tick. The mean perceived probabilities reported by respondents in Norway [18] and Sweden [19] were similar but slightly higher than that reported by respondents in Denmark (16 on a 100 point scale). We find similar differences in reported probabilities of contracting TBE if bitten by a tick. Respondents in Norway [14] and Sweden [12] again reported similar but higher probabilities than respondents in Denmark [10].

The responses regarding the perceived seriousness of a tick bite were rather similar across countries, with the average score for respondents from Norway and Sweden being 5.2 and 5.3 respectively on a 10 point scale. The higher average score for respondents in Denmark (5.8) indicates that these respondents found it more serious to get bit by a tick than did respondents in the other countries.

There was a larger difference between the country sub-samples in perceived seriousness of contracting LB. While respondents in Sweden reported a mean score of 7.2 (on a 0–10 scale), those in Denmark and Norway had significantly higher mean scores of 8.6 and 8.1, respectively. We also find that not only the mean values but also the distribution of the responses differ distinctly across the three countries. A higher share of the respondents in Denmark (43%) and Norway (34%) reported the highest value on the 0–10 scale of perceived seriousness compared with far fewer respondents in Sweden (17%). The perceived seriousness of contracting TBE was higher on average than for LB, with a mean of 9.0 on the 0–10 scale in the total sample. Respondents in Denmark reported a small but statistically significantly higher perceived seriousness (9.2) than respondents in Norway (8.7) and Sweden (9.0).

#### Knowledge

Turning to the degree of knowledge about tick-borne diseases, the general awareness of LB was high with 96% of the respondents stating that they had heard of the disease (see Table 1).^3^ However, this share was significantly lower in Norway, where 92% had heard of LB. Considerably fewer, 70% of all respondents, stated that they had heard of TBE but cross-country differences were considerable. Almost all respondents in Sweden reported having heard of TBE (97%) compared with Denmark (64%) and Norway (only 37%).

Respondents who reported having heard of LB and/or TBE were asked three knowledge questions related to each disease.^4^ The average score on the six knowledge questions was 3.2 out of six possible correct answers with significant differences between respondents in Norway (2.4), Denmark (3.0), and Sweden (4.1). Eighty-four per cent of the respondents knew that LB is not contagious, but the share was significantly lower in Norway than in the other countries. About half of all respondents thought that a vaccine against LB exists, which is incorrect. The respondents in Denmark did markedly better than others in this regard, with 59% giving the correct answer. About three in four of all respondents knew that LB is treatable with antibiotics. Again, a significantly lower share of the respondents in Norway answered correctly compared with those in Denmark and Sweden. Knowledge about TBE was considerably lower than about LB. As expected, knowledge about TBE was higher in Sweden than in Denmark and Norway. Eighty-five per cent of the respondents in Sweden knew that TBE is not contagious, compared with about half of respondents in Denmark and just one quarter of respondents in Norway. While a large majority of respondents in Sweden (77%) knew that there is a vaccine against TBE, far fewer respondents in Denmark (11%) and Norway (16%) answered this question correctly. Only 19% of all respondents knew that TBE cannot be treated with antibiotics. Also among respondents in Sweden, where TBE is relatively more prevalent, only 34% of the respondents answered this question correctly.

The average score on the picture identification task was 3.7 (scale 0–5) with minor variations across countries. The highest average score was found in Sweden followed by Norway and Denmark. For the two insect pictures, almost all respondents correctly identified these as non-ticks. Ninety-two per cent managed to correctly identify a blood-filled tick as a tick, whereas only about half of the respondents were correct on the other two tick pictures. From these answers it is clear that respondents struggled to identify ticks except when full of blood.

#### Differences in knowledge and risk perception across gender

To inform public policy, we also analysed differences in risk perceptions and knowledge between men and women and between different age groups. Table 2 reports differences in risk perceptions and knowledge about ticks and tick-borne diseases between female and male respondents.

**Table 2:** Gender differences in risk perceptions and knowledge related to ticks and tick-borne diseases

	[1]	[2]	[3]	[4]	[5]	[6]	[7]	*P*-values^b^
VARIABLES	Female	Male	All	sd^a^	min	max	N	H_0_:μ_1_ = μ_2_
Likelihood of being bitten by tick in next 12 months	33.21	30.95	32.13	31.85	0	100	2660	**0.068**
Likelihood of contracting LB if bitten by tick	21.07	15.42	18.44	19.51	0	100	2529	**0.000**
Likelihood of contracting TBE if bitten by tick	14.58	11.10	13.00	17.48	0	100	1822	**0.000**
Perceived seriousness of one tick bite	5.737	5.085	5.429	2.515	0	10	2632	**0.000**
Perceived seriousness of contracting LB	8.026	7.675	7.863	2.061	0	10	2538	**0.000**
Perceived seriousness of contracting TBE	9.229	8.792	9.032	1.574	0	10	1801	**0.000**
Ever heard of Borreliosis	0.979	0.947	0.964	0.186	0	1	2668	**0.000**
Ever heard of TBE	0.726	0.663	0.696	0.460	0	1	2668	**0.000**
Number of correctly identified tick pictures	3.728	3.730	3.729	1.114	0	5	2668	0.965
Number of correct answers to knowledge questions	3.457	3.028	3.253	1.642	0	6	2668	**0.000**

Notes: ^a^sd = The standard deviation for all respondents (column 3).

^b^ From Chi-squared test for dummy variables and an unpaired t-test for continuous variables. P-values < 0.10 are highlighted.

We find that female respondents on average reported significantly higher scores for all six risk perception questions. On average, female respondents also scored higher on the knowledge questions and a higher share of women had heard of LB and TBE. There were no significant differences between men and women in correctly identifying pictures of ticks.

#### Differences in knowledge and risk perception across age groups

Tables 3 and 4 reports differences in risk perceptions and knowledge about ticks and tick-borne diseases across age groups.

**Table 3:** Age differences in risk perceptions and knowledge related to ticks and tick-borne diseases

	[1]	[2]	[3]	[4]	[5]	[6]	[7]	[8]	[9]
VARIABLES	age1829	age3044	age4559	age60ormore	All	sd^a^	min	max	N
Likelihood of being bitten by tick in next 12 months	29.23	33.12	33.22	31.87	32.13	31.85	0	100	2660
Likelihood of contracting LB if bitten by tick	15.74	16.33	19.49	20.74	18.44	19.51	0	100	2529
Likelihood of contracting TBE if bitten by tick	11.65	11.21	13.98	14.30	13.00	17.48	0	100	1822
Perceived seriousness of one tick bite	4.593	5.052	5.586	6.062	5.429	2.515	0	10	2632
Perceived seriousness of contracting LB	7.647	7.698	7.876	8.100	7.863	2.061	0	10	2538
Perceived seriousness of contracting TBE	8.831	8.904	9.186	9.098	9.032	1.574	0	10	1801
Ever heard of Borreliosis	0.900	0.972	0.973	0.983	0.964	0.186	0	1	2668
Ever heard of TBE	0.603	0.696	0.699	0.742	0.696	0.460	0	1	2668
Number of correctly identified tick pictures	3.968	3.997	3.745	3.355	3.729	1.114	0	5	2668
Number of correct answers to knowledge questions	2.534	3.210	3.441	3.510	3.253	1.642	0	6	2668

**Table 4:** Analysis of statistically significant differences between age groups

VARIABLES	P-values^b^	P-values^b^	P-values^b^	P-values^b^	P-values^b^	P-values^b^
	H_0_:μ_1_ = μ_2_	H_0_:μ_1_ = μ3	H_0_:μ_1_ = μ4	H_0_:μ2 = μ3	H_0_:μ2 = μ4	H_0_:μ3 = μ4
Likelihood of being bitten by tick in next 12 months	0.269	0.249	0.983	1.000	1.000	1.000
Likelihood of contracting LB if bitten by tick	1.000	**0.016**	**0.000**	**0.016**	**0.000**	1.000
Likelihood of contracting TBE if bitten by tick	1.000	0.510	0.255	**0.081**	**0.022**	1.000
Perceived seriousness of one tick bite	**0.017**	**0.000**	**0.000**	**0.001**	**0.000**	**0.001**
Perceived seriousness of contracting LB	1.000	0.720	**0.003**	0.798	**0.001**	0.137
Perceived seriousness of contracting TBE	1.000	**0.024**	0.119	**0.096**	0.493	1.000
Ever heard of Borreliosis	**0.000**	**0.000**	**0.000**	0.963	0.151	0.168
Ever heard of TBE	**0.001**	**0.001**	**0.000**	0.902	**0.042**	**0.059**
Number of correctly identified tick pictures	1.000	**0.005**	**0.000**	**0.000**	**0.000**	**0.000**
Number of correct answers to knowledge questions	**0.000**	**0.000**	**0.000**	**0.041**	**0.002**	1.000

Notes: ^a^sd = The standard deviation for all respondents (column 5)

^b^ From Chi-squared test for dummy variables and an unpaired t-test for continuous variables. P-values < 0.10 are highlighted.

Older age groups have higher risk perceptions compared with younger age groups. This general pattern is consistent for all six risk perception questions (except for some small variations where those aged 45–59 have somewhat higher risk perceptions than the oldest age group) and several of the differences are also statistically significant. A higher share of respondents in the older age groups had heard of LB and TBE and score significantly higher on the six knowledge questions. The two older age groups scored significantly lower on the picture identification task.

### Associations between risk perceptions regarding tick bites, LB and TBE and other factors

Tables 5–7 show results for variables associated with the perceived seriousness of getting a single tick bite, LB and TBE, respectively, for all respondents in the sample (columns 1–3) as well as for each country separately (columns 4–6). In order to control for potential endogeneity between knowledge, exposure and risk perceptions, the analysis was performed in three steps. Column 1 reports associations with demographic variables only. The second column reports associations when variables related to general risk preferences and knowledge are included. The third column also includes associations with exposure related variables. For the country subsamples, associations are only reported when all explanatory variables are included in the model. Statistically significant associations at the 1, 5 and 10% levels are reported in the tables.

**Table 5:** Risk perception: Perceived seriousness of getting bitten by a tick. Regression results using OLS. Dependent variable: Perceived seriousness of getting bitten by a tick*

VARIABLES	[1]	[2]	[3]	[4]	[5]	[6]
	Scandinavia	Scandinavia	Scandinavia	DK^a^	NO^b^	SE^c^
Age 18–29	−0.469***	− 0.472***	−0.382***	− 0.820***	−0.239	− 0.293
	(0.151)	(0.150)	(0.147)	(0.297)	(0.264)	(0.220)
Age 45–59	0.502***	0.518***	0.490***	0.649***	0.312	0.499***
	(0.131)	(0.129)	(0.125)	(0.235)	(0.235)	(0.191)
Age 60–	0.950***	0.897***	0.813***	0.974***	0.576**	0.899***
	(0.126)	(0.125)	(0.123)	(0.229)	(0.234)	(0.188)
Female respondent	0.724***	0.652***	0.644***	0.263	0.485***	0.969***
	(0.097)	(0.097)	(0.095)	(0.179)	(0.177)	(0.144)
Has attended university	−0.313***	− 0.232**	− 0.227**	− 0.253	− 0.248	− 0.258*
	(0.106)	(0.105)	(0.102)	(0.213)	(0.193)	(0.147)
Norway	−0.518***	− 0.586***	− 0.608***			
	(0.125)	(0.123)	(0.120)			
Sweden	−0.549***	− 0.569***	− 0.486***			
	(0.117)	(0.139)	(0.136)			
General willingness to take risks		−0.196***	−0.181***	− 0.184***	− 0.272***	−0.104***
		(0.021)	(0.021)	(0.038)	(0.037)	(0.033)
Number of correct answers to Borrelia knowledge questions		−0.198***	− 0.140**	− 0.039	− 0.152	−0.209**
		(0.055)	(0.054)	(0.098)	(0.095)	(0.094)
Number of correct answers to TBE knowledge questions		−0.049	0.005	−0.132	− 0.013	0.127
		(0.059)	(0.059)	(0.116)	(0.109)	(0.095)
Has had one or more tick bites in lifetime			−1.158***	−1.095***	−1.136***	−1.087***
			(0.099)	(0.177)	(0.180)	(0.164)
Diagnosed with Lyme borreliosis ever			0.634***	0.929**	−0.626	0.822***
			(0.190)	(0.462)	(0.463)	(0.232)
Visits TBE areas at least monthly			0.188	0.012	0.331	0.232
			(0.134)	(0.307)	(0.320)	(0.169)
Constant	5.284***	6.718***	7.071***	7.114***	7.149***	5.860***
	(0.154)	(0.211)	(0.208)	(0.370)	(0.331)	(0.306)
Observations	2581	2581	2581	754	765	1062
R-squared	0.072	0.109	0.154	0.172	0.174	0.136

Notes: ^a^Denmark; ^b^Norway; ^c^Sweden

*The dependent variable is measured on a 0–10 scale where 0 equals ‘not serious as all’ and 10 equals ‘very serious’ in response to the question ‘How serious do you consider it is to be bitten by a tick?’

Standard errors in parentheses

*** *p* < 0.01, ** *p* < 0.05, * *p* < 0.1

**Table 6:** Risk perception: Perceived seriousness of getting Lyme borreliosis. Regression results using OLS. Dependent variable: perceived seriousness of getting Lyme borreliosis*

VARIABLES	[1]	[2]	[3]	[4]	[5]	[6]
	Scandinavia	Scandinavia	Scandinavia	DK^a^	NO^b^	SE^c^
Age 18–29	−0.005	− 0.039	−0.018	− 0.261	−0.177	0.134
	(0.127)	(0.127)	(0.127)	(0.230)	(0.233)	(0.201)
Age 45–59	0.120	0.153	0.161	0.071	0.053	0.293*
	(0.107)	(0.106)	(0.106)	(0.176)	(0.200)	(0.174)
Age 60–	0.323***	0.340***	0.364***	0.299*	0.399**	0.382**
	(0.103)	(0.103)	(0.104)	(0.173)	(0.197)	(0.172)
Female respondent	0.365***	0.365***	0.360***	0.377***	0.310**	0.385***
	(0.080)	(0.080)	(0.080)	(0.134)	(0.151)	(0.131)
Has attended university	−0.265***	− 0.187**	− 0.185**	− 0.316*	− 0.147	−0.160
	(0.088)	(0.088)	(0.088)	(0.162)	(0.169)	(0.134)
Norway	−0.424***	− 0.489***	− 0.481***			
	(0.104)	(0.103)	(0.103)			
Sweden	−1.395***	−1.136***	−1.095***			
	(0.096)	(0.115)	(0.116)			
General willingness to take risks		−0.070***	−0.066***	− 0.091***	− 0.102***	− 0.032
		(0.017)	(0.017)	(0.029)	(0.032)	(0.030)
Number of correct answers to Borrelia knowledge questions		−0.127***	−0.101**	0.083	−0.029	− 0.275***
		(0.049)	(0.049)	(0.078)	(0.090)	(0.088)
Number of correct answers to TBE knowledge questions		−0.227***	−0.201***	− 0.214**	−0.168**	− 0.159*
		(0.049)	(0.049)	(0.085)	(0.084)	(0.087)
Has had one or more tick bites in lifetime			−0.195**	0.079	−0.140	− 0.419***
			(0.085)	(0.134)	(0.155)	(0.149)
Diagnosed with Lyme borreliosis ever			−0.382**	−0.388	− 0.399	− 0.250
			(0.158)	(0.344)	(0.383)	(0.210)
Constant	8.425***	9.152***	9.162***	8.889***	8.705***	8.252***
	(0.128)	(0.180)	(0.182)	(0.290)	(0.299)	(0.282)
Observations	2488	2488	2488	731	710	1047
R-squared	0.098	0.118	0.123	0.058	0.048	0.054

Notes: ^a^Denmark; ^b^Norway; ^c^Sweden

*The dependent variable is measured on a 0–10 scale where 0 equals ‘not serious as all’ and 10 equals ‘very serious’ in response to the question ‘How serious do you consider it is to get the tick-borne disease Lyme borreliosis?’. Standard errors in parentheses

*** *p* < 0.01, ** p < 0.05, * p < 0.1

**Table 7:** Risk perception: Perceived seriousness of getting TBE. Regression results using OLS; Dependent variable: Perceived seriousness of getting TBE*

VARIABLES	[1]	[2]	[3]	[4]	[5]	[6]
	Scandinavia	Scandinavia	Scandinavia	DK^a^	NO^b^	SE^c^
Age 18–29	−0.007	0.033	0.018	0.095	−0.420	0.078
	(0.122)	(0.123)	(0.123)	(0.242)	(0.366)	(0.157)
Age 45–59	0.260**	0.243**	0.237**	0.184	0.577*	0.181
	(0.101)	(0.101)	(0.101)	(0.176)	(0.305)	(0.134)
Age 60–	0.198**	0.165*	0.150	0.212	0.241	0.082
	(0.096)	(0.097)	(0.098)	(0.171)	(0.289)	(0.132)
Female respondent	0.422***	0.387***	0.380***	0.308**	0.501**	0.365***
	(0.075)	(0.076)	(0.076)	(0.134)	(0.226)	(0.102)
Has attended university	0.092	0.081	0.092	−0.013	−0.103	0.186*
	(0.083)	(0.083)	(0.084)	(0.168)	(0.276)	(0.104)
Norway	−0.573***	−0.592***	− 0.578***			
	(0.120)	(0.123)	(0.123)			
Sweden	−0.185**	−0.247**	−0.260**			
	(0.087)	(0.101)	(0.102)			
General willingness to take risks		−0.037**	−0.037**	−0.070**	0.008	−0.037
		(0.017)	(0.017)	(0.028)	(0.047)	(0.023)
Number of correct answers to Borrelia knowledge questions		0.058	0.046	0.103	−0.145	0.078
		(0.047)	(0.047)	(0.082)	(0.129)	(0.067)
Number of correct answers to TBE knowledge questions		0.062	0.066	−0.106	0.330***	0.024
		(0.050)	(0.050)	(0.094)	(0.121)	(0.071)
Has had one or more tick bites in lifetime			0.072	0.218	−0.076	0.013
			(0.082)	(0.134)	(0.232)	(0.116)
Diagnosed with Lyme borreliosis ever			0.217	0.382	0.111	0.241
			(0.136)	(0.318)	(0.455)	(0.162)
Visits TBE areas at least monthly			−0.156*	−0.244	−0.289	−0.114
			(0.091)	(0.188)	(0.251)	(0.118)
Constant	8.792***	8.806***	8.805***	9.046***	8.193***	8.564***
	(0.122)	(0.173)	(0.176)	(0.294)	(0.461)	(0.223)
Observations	1769	1769	1769	483	253	1033
R-squared	0.038	0.043	0.047	0.053	0.082	0.035

Notes: ^a^Denmark; ^b^Norway; ^c^Sweden

*The dependent variable is measured on a 0–10 scale where 0 equals ‘not serious as all’ and 10 equals ‘very serious’ in response to the question ‘How serious do you consider it is to get the tick-borne disease TBE?”. Standard errors in parentheses

*** *p* < 0.01, ** p < 0.05, * *p* < 0.1

#### Risk perception regarding tick bites

Table 5 reports results for variables associated with the perceived seriousness of getting a tick bite.

Among demographic factors, increasing age and being female are positively and significantly associated with a higher risk perception for a single tick bite, while there is a significant negative association with educational attainment. Living in Denmark is positively and significantly associated with perceived seriousness. As expected, a greater willingness to take risks is negatively associated with perceived seriousness.

A higher score on the three knowledge questions about LB is significantly associated with lower perceived seriousness in the sample as a whole. In the regression analysis per country subsample, we only find significant associations between knowledge about LB and the perceived seriousness of a tick bite among respondents in Sweden. There are no significant associations between correct answers to the knowledge questions about TBE and perceived seriousness of a tick bite.

We find strong negative associations between ever having had a tick bite and high perceived seriousness of a tick bite. Having been diagnosed with LB, on the other hand, is positively associated with a high perceived seriousness. We find no significant associations between frequent visits to areas with TBE risk and the perceived seriousness of a tick bite.

#### Risk perception regarding LB

Table 6 reports the results for variables associated with perceived seriousness of getting LB. The structure of the table is similar to the structure of Table 5.

We find similar associations between several of the explanatory variables and high perceived seriousness regarding LB as with tick bites reported in Table 5. Women and people aged above 60 are on average significantly more likely than men and younger age groups to have high risk perceptions concerning LB. Educational attainment as well as a general greater willingness to take risks is negatively associated with high perceived seriousness. Living in Denmark is positively and significantly associated with higher perceived seriousness of LB.

Having more knowledge about LB as well as experiences with tick bites are negatively associated with perceived seriousness, but in the country subsamples these associations are only significant in Sweden. While knowledge about TBE was not significantly associated with risk perceptions concerning tick bites, we find a significant negative association between knowledge about TBE and the perceived seriousness of LB.

We find a negative association (significant in the full sample but not in the country subsamples) between having been diagnosed with LB and the perceived seriousness of the diseases.

#### Risk perception regarding TBE

Table 7 reports results for variables associated with the perceived seriousness of getting TBE. The structure of this table is similar to that of Tables 5 and 6.

Similar to our results concerning tick bite and LB risk perceptions, we find that being female, increasing age, living in Denmark and a general higher willingness to take risks are significantly associated with higher perceived seriousness of TBE.

While educational attainment and knowledge were significantly and negatively associated with higher perceived seriousness of a tick bite and LB, we find no similar associations with the perceived seriousness of TBE. On the contrary, we find a weak significant positive association between TBE risk perceptions and educational attainment in Sweden and number of correct answers to the knowledge questions about TBE among respondents in Norway.

We find no significant associations between perceived seriousness of TBE and exposure to tick bites, having been diagnosed with LB or visits to TBE risk areas.

## Discussion

Understanding risk perceptions is important since it is this perceived risk rather than the objective risk that determines behaviour [2, 3]. In this paper, we investigate risk perceptions and knowledge related to ticks, tick bites, LB and TBE in Denmark, Norway and Sweden.

### Risk perception

Risk perception can be divided into two parts, the probability of an event occurring and the severity of that event should it occur. Although survey respondents indicated that the probability of contracting TBE is generally lower than that of contracting LB, both these probabilities were grossly overrated compared with scientific estimates of actual probabilities. While one recent study found that around 2% of people in Sweden who had been bitten by a tick were diagnosed with LB [37], the probability of contracting LB after a tick bite stated by Swedish respondents in our study was around 20%. Similarly, the estimated probability of developing TBE after a tick bite is very high compared with existing prevalence studies in ticks [25, 38].

The perceptions of the seriousness of a single tick bite, of getting LB and of getting TBE were all high among the respondents, although respondents again were correct in estimating that contracting TBE is generally more severe than LB, as LB is treatable whereas TBE is not.

Our findings correspond to earlier research indicating that laypeople have higher risk perceptions concerning ticks and tick-borne diseases than experts [12, 13]. The properties of tick-related risks are possible explanations behind this gap, as they are relatively new, they have potentially serious health impacts, the probability of getting LB or TBE is very low, and the risk is difficult to control.

The associations we find between high risk perceptions and being female, being older and having lower educational attainment correspond to the associations found in several studies of risk perceptions in other areas [6] as well as with findings in earlier studies of risk perceptions specifically linked to tick-borne diseases in Sweden [13], Switzerland and Canada [12].

In line with an earlier study [13], we find that having had one or more tick bites is associated with a lower perceived seriousness of a tick bite. On the contrary, having been diagnosed with LB is positively associated with high risk perceptions for a tick bite. This indicates that experiencing tick bites leads to a downward adjustment of risk perceptions, unless you get LB.

While having been diagnosed with LB is positively associated with a higher perceived seriousness of a tick bite, it is negatively associated with the perceived seriousness of LB. Respondents reporting they have been diagnosed with LB (at some point in time) on average perceive LB as less serious than other respondents. A possible explanation for this is that while media is more likely to cover the serious or even extreme cases, most cases of LB actually give mild symptoms that disappear following antibiotic therapy [8]. Once you have actually experienced this mild type of LB, the perceived seriousness of the disease may hence be lower. Another potential explanation could be that if you are diagnosed with LB you are also given substantial information on the subject.

The high risk perceptions related to ticks and tick-borne diseases found in this study may have both positive and negative impacts on public health. On the one hand, they are likely to be positively associated with the use of protective measures against tick bites which may reduce actual disease risk [13–15, 17, 19]. On the other hand, high risk perceptions may negatively impact public health if they affect time spent on outdoor recreation, as such recreation has been found to be associated with various health benefits [39]. For example, one recent study finds that LB risk is negatively associated with time spent on outdoor recreational activities in the U.S [40].

### Knowledge

The average level of knowledge about ticks, LB and TBE was low among respondents. While most respondents had heard of LB, almost half of the respondents mistakenly believed there is a vaccine against LB and one in four did not know that LB can be treated with antibiotics.

In Sweden, where the incidence of TBE is considerably higher than in Denmark and Norway, almost all respondents had heard of this disease. However, the knowledge was not very substantial with only 34% knowing that TBE cannot be treated with antibiotics. Less than 20% of respondents in Denmark and Norway knew about the existence of TBE vaccines.

The respondents to our survey struggled to identify ticks when presented with a mix of pictures of ticks and insects. While 92% correctly identified a blood-filled tick, only about half of the respondents, managed to correctly identify a female adult tick or a tick nymph. As it is important to identify a tick before it attaches to the body and consequently before it is blood-filled [41], this calls for increased information.

### Information provision

Informing the general public and specific at-risk groups about tick bite prevention, disease symptoms and available treatments is a common public health strategy. A dual objective for public health policy is to induce precautionary behaviour, that can decrease the incidence of tick-borne diseases, without causing alarm leading to reductions in outdoor activities [42]. The large variation in knowledge about LB and especially about TBE among the respondents in this study indicates that information provision could be a relevant public health strategy. Furthermore, our finding that better knowledge about tick-borne disease is associated with a lower perceived seriousness of tick bites and LB and a higher perceived seriousness of TBE indicate that information provision could make risk perceptions more closely aligned with actual risk.

As men and younger age groups are on average less knowledgeable about LB and TBE, they may be particularly important to target with information about these diseases. However, information provision about ticks and tick-borne diseases is not always effective [21, 43]. In Denmark, where LB risk perceptions were significantly higher than in Norway and Sweden, knowledge about LB was also significantly higher but not negatively associated with LB risk perceptions. Thus, it is possible that risk perceptions in certain contexts are socially amplified through for example media [7]. In such contexts, risk communication focusing on helping the public understand probabilities and ‘get the numbers right’ will most likely need to be combined with participatory approaches aiming at enhancing trust in health authorities and the advice they provide [44–46]. One such initiative is a specialist centre for tick-borne diseases (*www*.*Flåttsenteret.no*) recently established in Norway with the aim of providing advice to the public and professionals on ticks and tick-borne diseases and establishing a dialogue with concerned citizens and patient groups.

### Limitations

Comparing our sample with the general population our sample is somewhat older, has a larger share of women and has a higher average level of educational attainment. As education tends to be associated with better knowledge and lower risk perception, it is possible that the level of knowledge is lower and risk perceptions higher in the population than our results indicate.

While our findings largely correspond with other studies on risk perceptions, the low share of variation explained in the regression analyses indicate that several other possible unmeasured factors may influence risk perceptions. As this is a cross-sectional study, several caveats, including that we cannot infer causality, apply to the interpretation and generalisation of our results.

There is also potential endogeneity in our study. Risk perceptions are clearly not independent from knowledge and behavioural variables. We address this problem by analysing associations between our risk perception variables and demographic variables separately from variables related to knowledge and behaviour.

Our three-country study also allowed us to compare knowledge and risk perceptions in different epidemiological settings, but where the relative cultural and political similarities among the Scandinavian countries reduce the potentially confounding factors that can be found in cross-country studies.

## Conclusion

There is a general lack of knowledge about ticks and tick-borne diseases. This concerns the recognition of ticks as well as the general knowledge about LB and TBE, including the availability of vaccines and antibiotic treatments. Risk perceptions are exaggerated regarding the probability of being infected by LB or TBE from a tick bite as well as regarding the severity of a tick bite and of contracting an LB infection.

Providing information that leads to increased knowledge about ticks and tick-borne diseases would therefore be a relevant public health strategy as it could make risk perceptions better aligned with actual risk. Should the TBE virus spread further in Denmark and Norway, information about TBE in general and the possibility of vaccination specifically would be especially important.

Overestimations of risk may negatively impact public health as it may lead to reduced time spent on outdoor recreation. Underestimation of risk may also negatively impact public health due to low use of protective measures and unnecessary exposure to tick bites and disease risk. A key challenge for public health authorities is to encourage precaution without causing alarm.

## Additional file

**Additional file 1.** Survey questionnaire on risk perceptions, knowledge and behaviour related to ticks and tick-borne diseases in Scandinavia.

## Data Availability

The dataset analyzed in this study as well as the questionnaire in the three languages are available in the repository of the Swedish National Data Service, https://snd.gu.se/en/catalogue/study/snd1119, 10.5878/3qmz-r537. An English translation of the questionnaire is included in appendix.

## Abbreviations

LB: Lyme borreliosis
TBE: Tick-borne encephalitis
VIF: variance inflation factor

## Footnotes

1 An English translation of the questionnaire – which was distributed in Danish, Norwegian and Swedish – is included as Additional file 1 to this article.

2 The share of the population with internet access was 94% in Denmark and Sweden and 97% in Norway in 2016, which means that web-based surveys exclude only a very small share of the population in Scandinavia.

3 For the questions “Have you ever heard of LB?” and “Have you ever heard of TBE?” we classify those who respond “Don’t know” to the question as “No”. These answers represent very small shares of our sample (0.5 and 3.6% for LB and TBE, respectively).

4 We assume that those who had not heard of the disease do not know any facts about the disease. Those who answered that they have not heard of LB or TBE were classified as not having a correct answer to the knowledge questions.

## References

[1] Brewer NT, Chapman GB, Gibbons FX, Gerrard M, McCaul KD, Weinstein ND (2007). Meta-analysis of the relationship between risk perception and health behavior: the example of vaccination. Health Psychol.

[2] Ferrer RA, Klein WMP (2015). Risk perceptions and health behavior. Curr Opin Psychol.

[3] Viscusi WK (1990). Do Smokers Underestimate Risk?. J Pol Econ.

[4] Fischhoff B, Slovic P, Lichtenstein S, Read S, Combs B (1978). How safe is safe enough? A psychometric study of attitudes towards technological risks and benefits. Integrating Knowl Pract Adv Hum Dignity.

[5] Loewenstein GF, Hsee CK, Weber EU, Welch N (2001). Risk as Feelings. Psychol Bull.

[6] Slovic P (1987). Perception of risk. Science.

[7] Kasperson RE, Renn O, Slovic P, Brown HS, Emel J, Goble R (1988). The social amplification of risk: a conceptual framework. Risk Anal.

[8] Stanek G, Wormser GP, Gray J, Strle F (2012). Lyme borreliosis. Lancet.

[9] Lindquist Lars, Vapalahti Olli (2008). Tick-borne encephalitis. The Lancet.

[10] Haglund M, Günther G (2003). Tick-borne encephalitis - pathogenesis, clinical course and long-term follow-up. Vaccine.

[11] Kaiser R (1999). The clinical and epidemiological profile of tick-borne encephalitis in southern Germany 1994-98 - a prospective study of 656 patients. Brain.

[12] Aenishaenslin C, Ravel A, Michel P, Gern L, Milord F, Waaub JP (2014). From Lyme disease emergence to endemicity: a cross sectional comparative study of risk perceptions in different populations. BMC Public Health.

[13] Slunge Daniel, Boman Anders (2018). Learning to live with ticks? The role of exposure and risk perceptions in protective behaviour against tick-borne diseases. PLOS ONE.

[14] Aenishaenslin C, Michel P, Ravel A, Gern L, Milord F, Waaub J-P (2015). Factors associated with preventive behaviors regarding Lyme disease in Canada and Switzerland: a comparative study. BMC Public Health.

[15] Beaujean DJMA, Bults M, van Steenbergen JE, Voeten HACM (2013). Study on public perceptions and protective behaviors regarding Lyme disease among the general public in the Netherlands: implications for prevention programs. BMC Public Health.

[16] Herrington JE (2004). Risk perceptions regarding ticks and Lyme disease: a national survey. Am J Prev Med.

[17] Jepsen MT, Jokelainen P, Jore S, Boman A, Slunge D, Krogfelt KA. Protective practices against tick bites in Denmark, Norway and Sweden: a questionnaire-based study BMC Publ Health. 2019;Under review. PUBH-D-19-00214R2. 10.1186/s12889-019-7613-4 PMC6805683 31640665

[18] Jones TF, Garman RL, LaFleur B, Stephan SJ, Schaffner W (2002). Risk factors for tick exposure and suboptimal adherence to preventive recommendations. Am J Prev Med.

[19] Mowbray F, Amlôt R, Rubin GJ (2014). Predictors of protective behaviour against ticks in the UK: a mixed methods study. Ticks Tick-Borne Dis.

[20] Shadick NA, Daltroy LH, Phillips CB, Liang US, Liang MH (1997). Determinants of tick-avoidance behaviors in an endemic area for Lyme disease. Am J Prev Med.

[21] Gupta S, Eggers P, Arana A, Kresse B, Rios K, Brown L (2018). Knowledge and preventive behaviors towards tick-borne diseases in Delaware. Ticks Tick-Borne Dis.

[22] Lindsay LR, Ogden NH, Schofield SW (2015). Review of methods to prevent and reduce the risk of Lyme disease. Can Commun Dis Rep.

[23] Jaenson T, Jaenson DG, Eisen L, Petersson E, Lindgren E (2012). Changes in the geographical distribution and abundance of the tick Ixodes ricinus during the past 30 years in Sweden. Parasit Vectors.

[24] Jore S, Viljugrein H, Hofshagen M, Brun-Hansen H, Kristoffersen AB, Nygård K (2011). Multi-source analysis reveals latitudinal and altitudinal shifts in range of Ixodes ricinus at its northern distribution limit. Parasit Vectors.

[25] Pettersson JH-O, Golovljova I, Vene S, Jaenson TG (2014). Prevalence of tick-borne encephalitis virus in Ixodes ricinus ticks in northern Europe with particular reference to southern Sweden. Parasit Vectors.

[26] Wilhelmsson P, Fryland L, Lindblom P, Sjöwall J, Ahlm C, Berglund J (2016). A prospective study on the incidence of Borrelia burgdorferi sensu lato infection after a tick bite in Sweden and on the Åland Islands, Finland (2008–2009). Ticks Tick-Borne Dis.

[27] Butler AD, Sedghi T, Petrini JR, Ahmadi R (2016). Tick-borne disease preventive practices and perceptions in an endemic area. Ticks Tick-Borne Dis.

[28] Phillips CB, Liang MH, Sangha O, Wright EA, Fossel AH, Lew RA, et al. Lyme disease and preventive behaviors in residents of Nantucket Island. Massachusetts Am J Prev Med. 2001;20(3):219–24. 10.1016/s0749-3797(00)00315-9 11275450

[29] Stjernberg L, Berglund J (2005). Tick prevention in a population living in a highly endemic area. Scand J Public Health.

[30] Zöldi V, Turunen T, Lyytikäinen O, Sane J (2017). Knowledge, attitudes, and practices regarding ticks and tick-borne diseases, Finland. Ticks Tick-Borne Dis.

[31] Brewer Noel T., Weinstein Neil D., Cuite Cara L., Herrington James E. (2004). Risk perceptions and their relation to risk behavior. Annals of Behavioral Medicine.

[32] Dohmen T, Falk A, Huffman D, Sunde U, Schupp J, Wagner G (2011). Individual risk attitudes: measurement, determinants, and behavioral consequences. J Eur Econ Assoc.

[33] Manski CF (2004). Measuring expectations. Econometrica.

[34] Statistics Denmark. 2016 [Available from: www.dst.dk.

[35] Statistics Norway. 2016 [Available from: www.ssb.no.

[36] Statistics Sweden. 2016 [Available from: www.scb.se.

[37] Wilhelmsson P, Lindblom P, Fryland L, Ernerudh J, Forsberg P, Lindgren P-E (2013). Prevalence, diversity, and load of Borrelia species in ticks that have fed on humans in regions of Sweden and Åland Islands, Finland with different Lyme borreliosis incidences. PLoS One.

[38] Lindblom P, Wilhelmsson P, Fryland L, Sjöwall J, Haglund M, Matussek A (2014). Tick-borne encephalitis virus in ticks detached from humans and follow-up of serological and clinical response. Ticks Tick-Borne Dis.

[39] Williams F. The nature fix: why nature makes us happier, healthier, and more creative: Amazon; 2017.

[40] Berry Kevin, Bayham Jude, Meyer Spencer R., Fenichel Eli P. (2017). The Allocation of Time and Risk of Lyme: A Case of Ecosystem Service Income and Substitution Effects. Environmental and Resource Economics.

[41] Corapi Kristin M, White Marc I, Phillips Charlotte B, Daltroy Lawren H, Shadick Nancy A, Liang Matthew H (2007). Strategies for primary and secondary prevention of Lyme disease. Nature Clinical Practice Rheumatology.

[42] Quine CP, Barnett J, Dobson ADM, Marcu A, Marzano M, Moseley D (2011). Frameworks for risk communication and disease management: the case of Lyme disease and countryside users. Philos Trans R Soc B.

[43] Mowbray F, Amlôt R, Rubin GJ (2012). Ticking all the boxes? A systematic review of education and communication interventions to prevent tick-borne disease. Vector-Borne Zoonotic Dis.

[44] Fischhoff B (1995). Risk perception and communication unplugged - 20 years of process. Risk Anal.

[45] Fischhoff B, Scheufele DA (2013). The science of science communication. Proc Natl Acad Sci.

[46] Renn O, Klinke A, Asselt M (2011). Coping with complexity, uncertainty and ambiguity in risk governance: a synthesis. A J Hum Environ.

