# Supplementary material for: Who is afraid of ticks and tick-borne diseases? Results from a cross-sectional survey in Scandinavia
Source: BMC Public Health. 2019 Dec 11;19:1666. doi: 10.1186/s12889-019-7977-5 (PMC6907266; doi:10.1186/s12889-019-7977-5)
Supplement: Supplementary file 1 — Additional file 1. Survey questionnaire on risk perceptions, knowledge and behaviour related to ticks and tick-borne diseases in Scandinavia. [file 12889_2019_7977_MOESM1_ESM.pdf]

Supplementary material to the article "*Who is afraid of ticks and tick-borne diseases? Results from a cross-sectional survey in Scandinavia*" by Slunge et al.

Survey questionnaire on risk perceptions, knowledge and behaviour related to ticks and tick-borne diseases

[This is an English translation of the questionnaire in Swedish which was sent out to respondents in Sweden in October 2016. Similar surveys were sent out simultaneously in Danish and Norwegian to respondents in Denmark and Norway. Please note that this was a web-based questionnaire. Routing was used so that all respondents were not presented with all the questions. The questionnaire was translated by a professional language editor contracted by the University of Gothenburg]

[qYear_ – STRING – single – Must answer]
What year were you born? <i>Please respond in the field below and choose the correct year from the list</i>

[qGender – CATEGORICAL – single – Must answer]
Gender:

- (_1) Woman
- (_2) Man
- (_3) Other
- (_4) I choose not to respond

[qMunicipality – STRING – single – Must answer]
In what municipality do you live? <i> Please respond in the field below and choose the correct municipality from the list</i>

[qZip – STRING – single – Must answer]
What is your postal code? <i> Please respond in the field below and choose the correct postal code from the list </i>

[q1 – GRID – single – Must answer]
Next, we will show you a series of pictures. Which pictures do you believe show a tick? <i>Please provide one response per picture.</i>

	Yes	No	Don't know
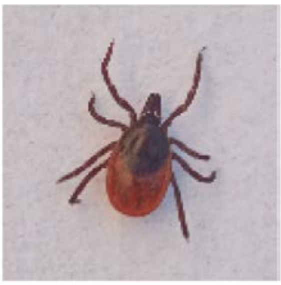	◯	◯	◯
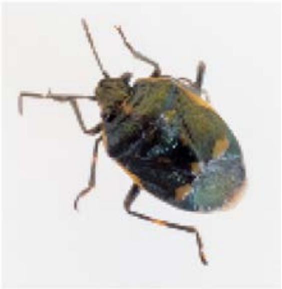	◯	◯	◯
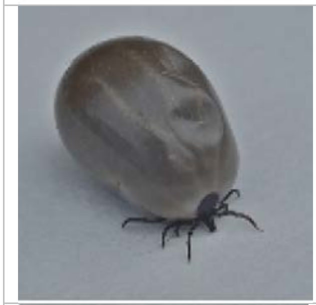	◯	◯	◯
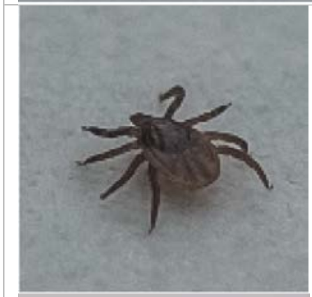 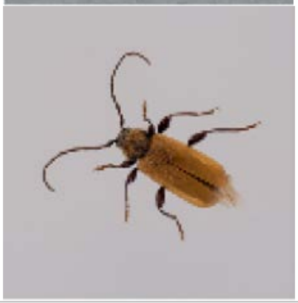	◯	◯	◯

[q2 – CATEGORICAL – single – Must answer]
Have you ever heard of Lyme borreliosis?

- (_1) Yes
- (_2) No
- (_3) Don't know

[q3 – CATEGORICAL – single – Must answer]
Have you ever heard of TBE (tick-borne encephalitis)?

- (_1) Yes
- (_2) No
- (_3) Don't know

[If q2 = {_1} Then q4.QuestionFilter = q4.QuestionFilter + {_1,_2,_3}]

[If q3 = {_1} Then q4.QuestionFilter = q4.QuestionFilter + {_4,_5,_6}]

[q4 – GRID – single – Must answer]
Which of the following statements do you believe are correct? <i> Please provide one response per statement </i> {#ex_script}

	True	False	Don't know
**Lyme borreliosis can spread from one person to another**	◯	◯	◯
**You can get vaccinated against Lyme borreliosis**	◯	◯	◯
**Lyme borreliosis can be treated with antibiotics**	◯	◯	◯
**TBE (tick-borne encephalitis) can spread from one person to another**	◯	◯	◯
**You can get vaccinated against TBE (tick-borne encephalitis)**	◯	◯	◯
**TBE (tick-borne encephalitis) can be treated with antibiotics**	◯	◯	◯

[q6 – CATEGORICAL – single – Must answer]
Have you ever been bit by a tick?

- (_1) Yes
- (_2) No
- (_3) Don't know

[If q6 = {_1} Then]

[q8_ – CATEGORICAL – multiple – Must answer]
Where geographically did your get a tick bite? <i>Multiple responses possible.</i>

- (_1) In the municipality where I currently live
- (_2) Somewhere else in Sweden
- (_3) In another country
- (_4) Don't know

[q8a_ – CATEGORICAL – multiple – Must answer]
Where else in Sweden have you been bit by a tick? Multiple responses possible. You can list up to 10 municipalities where you have been bit by a tick. If you have been bit by a tick in more than 10 Swedish municipalities, please report the 10 most recent municipalities where you have been bit by a tick (besides the municipality where you currently live).

- (_1) Municipality:
- (_2) Municipality:
- (_3) Municipality:
- (_4) Municipality:
- (_5) Municipality:
- (_6) Municipality:
- (_7) Municipality:
- (_8) Municipality:
- (_9) Municipality:
- (_10) Municipality:
- (_16) Don't know

[q8b_ – CATEGORICAL – multiple – Must answer]
In what other countries have you been bit by a tick? Multiple responses possible. You can list up to three countries (besides Sweden) where you have been bit by a tick. If you have been bit by a tick in more than three countries, please report the three most recent countries besides Sweden where you have been bit by a tick. (_1) Country:

- (_2) Country:
- (_3) Country:
- (_16) Don't know

[q7 – CATEGORICAL – single – Must answer]
How many times have you been bit by a tick in <i>the last 12 months </i>? <i>It may be difficult to remember all bites. Please respond to the best of your ability.</i>

[If q6.Response.Value.ContainsAny({_1,_3}) Then]

[q9 – CATEGORICAL – single – Must answer]
Have you had a tick-borne disease in the last 12 months?

- (_1) Yes
- (_2) No
- (_3) Don't know

[If q9 = {_1} Then]

[q10_ – CATEGORICAL – multiple – Must answer]
What disease(s)? (Multiple responses possible)

- (_1) Lyme borreliosis
- (_2) TBE (tick-borne encephalitis)
- (_3) Other disease (please specify):
- (_4) Don’t know

[If q10_.Response.Value.ContainsAny({_1}) Then]

[q11a – CATEGORICAL – single – Must answer]
Did a doctor conform that it was Lyme borreliosis?

- (_1) Yes
- (_2) No
- (_3) Don't know

[If q10_.Response.Value.ContainsAny({_2}) Then]

[q11b – CATEGORICAL – single – Must answer]
Did a doctor conform that it was TBE (tick-borne encephalitis)?

- (_1) Yes
- (_2) No
- (_3) Don't know

[If q10_.Response.Value.ContainsAny({_3}) Then]

[q11c – CATEGORICAL – single – Must answer]
Was the illness you specified as “Other disease” confirmed by a doctor?

- (_1) Yes
- (_2) No
- (_3) Don't know

[If q9.Response.Value.ContainsAny({_2,_3}) Then]

[qx – CATEGORICAL – single – Must answer]
Have you ever had a tick-borne disease?

- (_1) Yes
- (_2) No
- (_3) Don't know

[If qx = {_1} Then]

[qy_ – CATEGORICAL – multiple – Must answer]
What disease(s)? <i> Multiple responses possible</i>

- (_1) Lyme borreliosis
- (_2) TBE (tick-borne encephalitis)
- (_3) Other disease (please specify):

- (_4) Don’t know

[If qy_.Response.Value.ContainsAny({_1}) Then]

[qy1 – CATEGORICAL – single – Must answer]
Did a doctor conform that it was Lyme borreliosis?

- (_1) Yes
- (_2) No
- (_3) Don't know

[If qy_.Response.Value.ContainsAny({_2}) Then]

[qy2 – CATEGORICAL – single – Must answer]
Did a doctor conform that it was TBE (tick-borne encephalitis)?

- (_1) Yes
- (_2) No
- (_3) Don't know

[If qy_.Response.Value.ContainsAny({_3}) Then]

[qy3 – CATEGORICAL – single – Must answer]
Was the illness you specified as “Other disease” confirmed by a doctor?

- (_1) Yes
- (_2) No
- (_3) Don't know

[q5 – CATEGORICAL – single – Must answer]
On average, how often do you see a tick <i>from May to September</i>?

- (_1) Daily
- (_2) Weekly
- (_3) Monthly
- (_4) Less often than every month
- (_5) I have never seen a tick
- (_6) Don't know

[q12 – CATEGORICAL – single – Must answer]
Do you have one or more children under age 18?

- (_1) Yes
- (_2) No
- (_3) I choose not to respond

[If q12 = {_1} Then]

[q13 – CATEGORICAL – single – Must answer]
Has your child ever been bit by a tick? <i>If you have more than one child, please think only of your oldest child under age 18 when you respond.</i>

- o (_1) Yes
- o (_2) No
- o (_3) Don't know

[If q13 = {_1} Then]

[q14 – CATEGORICAL – single – Must answer]
How many times have your child been bit by a tick in the last 12 months? <i> If you have more than one child, please think only of your oldest child under age 18 when you respond. It may be difficult to remember exactly. Please respond to the best of your ability.</i>

[q15 – CATEGORICAL – single – Must answer]
Has your child had a tick-borne disease in the past 12 months? <i> If you have more than one child, please think only of your oldest child under age 18 when you respond.</i>

- o (_1) Yes
- o (_2) No
- o (_3) Don't know

[If q15 = {_1} Then]

[q16_ – CATEGORICAL – multiple – Must answer]
What disease(s)? <i> Multiple responses possible.</i>

- ☐ (_1) Lyme borreliosis
- ☐ (_2) TBE (tick-borne encephalitis)
- ☐ (_3) Other disease (please specify):
- ☐ (_4) Don’t know

[If q16_.Response.Value.ContainsAny({_1}) Then]

[q17a – CATEGORICAL – single – Must answer]
Did a doctor conform that it was Lyme borreliosis?

- o (_1) Yes
- o (_2) No
- o (_3) Don't know

[If q16_.Response.Value.ContainsAny({_2}) Then]

[q17b – CATEGORICAL – single – Must answer]
Did a doctor conform that it was TBE (tick-borne encephalitis)?

- o (_1) Yes
- o (_2) No
- o (_3) Don’t know

[If q16_.Response.Value.ContainsAny({_3}) Then]

[q17c – CATEGORICAL – single – Must answer]
Was the illness you specified as “Other disease” confirmed by a doctor?

- (_1) Yes
- (_2) No
- (_3) Don't know

[If q15.Response.Value.ContainsAny({_2,_3}) Then]

[qz – CATEGORICAL – single – Must answer]
Has your child <i> ever had a tick-borne disease? <i> If you have more than one child, please think only of your oldest child under age 18 when you respond. </i>

- (_1) Yes
- (_2) No
- (_3) Don't know

[If qz = {_1} Then]

[qh_ – CATEGORICAL – multiple – Must answer]
What disease(s)? <i> Multiple responses possible.</i>

- ☐ (_1) Lyme borreliosis
- ☐ (_2) TBE (tick-borne encephalitis)
- ☐ (_3) Other disease (please specify):
- ☐ (_4) Don’t know

[If qh_.Response.Value.ContainsAny({_1}) Then]

[qh1 – CATEGORICAL – single – Must answer]
Did a doctor conform that it was Lyme borreliosis?

- (_1) Yes
- (_2) No
- (_3) Don't know

[If qh_.Response.Value.ContainsAny({_2}) Then]

[qh2 – CATEGORICAL – single – Must answer]
Did a doctor conform that it was TBE (tick-borne encephalitis)?

- (_1) Yes
- (_2) No
- (_3) Don't know

[If qh_.Response.Value.ContainsAny({_3}) Then]

[qh3 – CATEGORICAL – single – Must answer]
Was the illness you specified as “Other disease” confirmed by a doctor?

- (_1) Yes
- (_2) No
- (_3) Don't know

[q18_ – CATEGORICAL – multiple – Must answer]
Do you have any pets that spend time outside? <i> Multiple responses possible.</i>

- (_1) No
- (_2) Yes, cat
- (_3) Yes, dog
- (_4) Yes, other animal

[If q18_.Response.Value.ContainsAny({_2,_3,_4}) Then]

[q19 – CATEGORICAL – single – Must answer]
How often have you seen ticks on your pet(s) from May to September?

- (_1) Daily
- (_2) Weekly
- (_3) Monthly
- (_4) Less often than every month
- (_5) Never

[If q18_.Response.Value.ContainsAny({_2,_3,_4}) Then q20.QuestionFilter = q20.QuestionFilter + {_7}]

[q20 – GRID – single – Must answer]
To what extent do you take any of the following actions <i>to protect yourself against tick bites</i> when spending time in the outdoors or in other places where you may be exposed to ticks?
<i>Please give one response per suggested action.</i> {#ex_script}

	Never	Rarely	Often	Always
I wear clothes that cover my entire legs and arms	○	○	○	○
I use mosquito or tick repellants	○	○	○	○
I tuck my trousers into my socks	○	○	○	○
I avoid tall grass and walking near bushes	○	○	○	○
I check my body and clothes for ticks when visiting areas where I may be exposed to them	○	○	○	○
I check my body for ticks after visiting areas where I may have been exposed to them	○	○	○	○
I check my pet(s) for ticks	○	○	○	○

[If q12 = {_1} Then]

[q21 – CATEGORICAL – single – Must answer]
How often do you check your child's/children's bodies for ticks after spending time in the outdoors or elsewhere where they may have been exposed to ticks?

- o (_1) Never
- o (_2) Rarely
- o (_3) Often
- o (_4) Always

[If q18_.Response.Value.ContainsAny({_2,_3,_4}) Then q22.QuestionFilter = q22.QuestionFilter + {_7}]

[q22 – GRID – single – Must answer]
What level of protection against tick bites and tick-borne diseases do you believe the following actions provide? <i>Please provide one response per action.</i>{#ex_script}

	No protection	Weak protection	Fairly strong protection	Very strong protection
**Wearing clothes that cover one's entire legs and arms**	◯	◯	◯	◯
**Using mosquito or tick repellants**	◯	◯	◯	◯
**Tucking trousers into socks**	◯	◯	◯	◯
**Avoiding tall grass and not walking near bushes**	◯	◯	◯	◯
**Checking one's body and clothes for ticks when being outside**	◯	◯	◯	◯
**Checking one's body for ticks after having been outside**	◯	◯	◯	◯
**Checking pets for ticks**	◯	◯	◯	◯

[If q2 = {_1} Then sQ23 = sQ23 + "q23B,"]

[If q3 = {_1} Then sQ23 = sQ23 + "q23C,"]

[q23_intro – INFO – single – Must answer]
How serious do you believe it is to: <i>Please respond on a scale from 0 to 10, where 0 means = "not serious at all" and 10 = "very serious".</i>

[q23A – GRID – single – Must answer]
How serious do you believe it is to get bit by a tick? <i> Please respond on a scale from 0 to 10, where 0 = "not serious at all" and 10 = "very serious".</i>

	0 = Not serious at all	1	2	3	4	5	6	7	8	9	10 = Very serious	Don't know
	◯	◯	◯	◯	◯	◯	◯	◯	◯	◯	◯	◯

[q23B – GRID – single – Must answer]
How serious do you believe it is to get the tick-borne disease called Lyme borreliosis? <i> Please respond on a scale from 0 to 10, where 0 = "not serious at all" and 10 = "very serious".</i>

	0 = Not serious at all	1	2	3	4	5	6	7	8	9	10 = Very serious	Don't know
	◯	◯	◯	◯	◯	◯	◯	◯	◯	◯	◯	◯

[q23C – GRID – single – Must answer]
How serious do you believe it is to get the tick-borne disease called TBE (tick-borne encephalitis)? <i> Please respond on a scale from 0 to 10, where 0 = "not serious at all" and 10 = "very serious".</i>

	0 = Not serious at all	1	2	3	4	5	6	7	8	9	10 = Very serious	Don't know
	◯	◯	◯	◯	◯	◯	◯	◯	◯	◯	◯	◯

[END PAGE – q23_page]

[q24 – INFO – single – Optional]
How likely are you to get bit by a tick in the next 12 months? <i>Please estimate the likelihood in per cent from 0 to 100 and write the value in the field below, where 0 = "I'm absolutely certain I will not get bit" and 100 = "I'm absolutely certain I will get bit".</i>

- (NotMust)

[If q2 = {_1} Then]

[q25 – INFO – single – Optional]
If you were to get bitten by a tick, how likely do you think you would be to get Lyme borreliosis?

<i> Please estimate the likelihood in per cent from 0 to 100 and write the value in the field below, where 0 = “I’m absolutely certain I would not get Lyme borreliosis” and 100 = “I’m absolutely certain I would get Lyme borreliosis”.</i>

- (NotMust)

[If q3 = {_1} Then]

[q26 – INFO – single – Optional]
If you were to get bitten by a tick, how likely do you think you would be to get TBE (tick-borne encephalitis)? <i> Please estimate the likelihood in per cent from 0 to 100 and write the value in the field below, where 0 = “I’m absolutely certain I would not get TBE” and 100 = “I’m absolutely certain I would get TBE”'</i>

- (NotMust)

[q27 – GRID – single – Must answer]
How would you describe your overall willingness to take risks? <i> Please respond on a scale from 0 to 10, where 0 = "not willing to take risks at all" and 10 ="very willing to take risks".</i>

	0 = Not willing to take risks at all	1	2	3	4	5	6	7	8	9	10 = Very willing to take risks

[q31 – CATEGORICAL – single – Must answer]
Have you been vaccinated against TBE (tick-borne encephalitis)?

- (_1) Yes
- (_2) No
- (_3) Don't know

[If q31 = {_1} Then]

[q32 – CATEGORICAL – single – Must answer]
What year did you receive your most recent injection of the TBE (tick-borne encephalitis) vaccine? <i> This may be difficult to remember exactly. Please respond to the best of your ability.</i>

- (_1) Year:
- (_2) Don't know/can’t remember

[q33 – CATEGORICAL – single – Must answer]
How many doses of the TBE (tick-borne encephalitis) vaccine have you received in total? <i> This may be difficult to remember exactly. Please respond to the best of your ability.</i>

- (_1) Number of doses:
- (_2) Don't know/can’t remember

[If q3 = {_1} Then]

[q34 – CATEGORICAL – single – Must answer]
Ticks that carry the TBE virus are present in some areas. From May to September, how often do you on average spend time in areas where you know or have heard there is a risk of exposure to ticks that carry the TBE virus</i>?

- (_1) More often than 3 times a week
- (_2) 1–3 times a week
- (_3) 1–3 times a month
- (_4) Less often
- (_5) Never
- (_6) Don't know

[q35 – CATEGORICAL – single – Must answer]
Have you ever tried to find information about ticks and/or tick-borne diseases? <i>Please choose one response option.</i>

- (_1) Yes, in the last 12 months
- (_2) Yes, but not in the last 12 months
- (_3) No
- (_4) Don't know

[If q35.Response.Value.ContainsAny({_1,_2}) Then]

[q36_ – CATEGORICAL – multiple – Must answer]
From which of the sources listed below have you tried to acquire information about ticks and tick-borne diseases? <i>Please mark as many sources as you want.</i>

- ☐ (_1) Your doctor
- ☐ (_2) The national healthcare telephone hotline(*Sjukvårdsupplysningen*)
- ☐ (_3) The national health agency
- ☐ (_4) A pharmacy
- ☐ (_5) Wikipedia
- ☐ (_6) Internet
- ☐ (_7) Social media (for example Facebook)
- ☐ (_8) Newspapers and magazines
- ☐ (_9) Radio
- ☐ (_10) TV
- ☐ (_11) Immunisation providers
- ☐ (_12) Friends
- ☐ (_13) Other sources:
- ☐ (_14) Don't know/Can’t remember

[q37_ – CATEGORICAL – multiple – Must answer]
What type of information have you looked for? <i> Multiple responses possible.</i>

- ☐ (_1) Information about in which areas the TBE virus is present
- ☐ (_2) Information about in which areas the bacteria that causes Lyme borreliosis is present
- ☐ (_3) Information about in which areas ticks are present
- ☐ (_4) Information about how to best remove a tick after a bite
- ☐ (_5) Information about how to avoid tick bites
- ☐ (_6) Information about symptoms of Lyme borreliosis
- ☐ (_7) Information about symptoms of TBE
- ☐ (_8) Information about the treatment of Lyme borreliosis
- ☐ (_9) Information about the treatment of TBE
- ☐ (_10) Information about TBE vaccination
- ☐ (_11) Other information:
- ☐ (_12) Don't know/Can't remember

[If q35.Response.Value.ContainsAny({_3,_4}) Then]

[q38_ – CATEGORICAL – multiple – Must answer]
If you were to look for information, what type of information about ticks and tick-borne diseases would you be the most interested in? <i> Multiple responses possible.</i>

- ☐ (_1) Information about in which areas the TBE (tick-borne encephalitis) virus is present
- ☐ (_2) Information about in which areas the bacteria that causes Lyme borreliosis is present
- ☐ (_3) Information about in which areas ticks are present
- ☐ (_4) Information about how to best remove a tick after a bite
- ☐ (_5) Information about how to avoid tick bites
- ☐ (_6) Information about symptoms of Lyme borreliosis
- ☐ (_7) Information about symptoms of TBE (tick-borne encephalitis)
- ☐ (_8) Information about the treatment of Lyme borreliosis
- ☐ (_9) Information about the treatment of TBE (tick-borne encephalitis)
- ☐ (_10) Information about TBE (tick-borne encephalitis) vaccination
- ☐ (_11) Other information:
- ☐ (_12) Don't know
- ☐ (_13) I'm not interested in information about ticks and tick-borne diseases

[q40 – GRID – single – Must answer]
How would you describe your overall health? <i> Please respond on a scale from 0 to 10, where 0 = "very poor" and 10 = "very good".</i>

	0 = Very poor	1	2	3	4	5	6	7	8	9	10 = Very good

[q41 – GRID – single – Must answer]
In your opinion, to what degree can you trust: {#ex_script11} <i> Please respond on a scale from 0 to 10, where 0 = "to a very low degree" and 10 = "to a very high degree".</i>

	0 = To a very low degree	1	2	3	4	5	6	7	8	9	10 = To a very high degree
**People in general**	○	○	○	○	○	○	○	○	○	○	○
**Information from healthcare providers**	○	○	○	○	○	○	○	○	○	○	○
**Recommendations from healthcare providers about vaccinations**	○	○	○	○	○	○	○	○	○	○	○

[q42 – CATEGORICAL – single – Must answer]
What is your highest completed level of education?

- (_1) Less than compulsory school
- (_2) Compulsory school
- (_3) Upper secondary school
- (_4) University, 1–3 years (in Sweden, a Bachelor's degree is typically awarded upon completion of a 3-year programme)
- (_5) University, more than 3 years
- (_6) Doctoral studies
- (_7) I choose not to respond

[q43 – CATEGORICAL – single – Must answer]
What is your HOUSEHOLD'S total after-tax income per month? <i> Please estimate your household's total after-tax monthly income from all sources, such as salaries, pension, unemployment benefit and child allowance.</i>

- (_1)
- (_2) I choose not to respond

[q28 – GRID – single – Must answer - Randomized]
On average, how often do you engage in the following activities <i>from May to September</i>?
<i>Please provide one response per activity </i>

	More than 3 times/week	1–3 times/week	1–3 times/month	Less often	Never
Hiking/running in the outdoors	○	○	○	○	○
Wild berry and mushroom picking	○	○	○	○	○
Bicycling in the outdoors	○	○	○	○	○
Horseback riding in the outdoors	○	○	○	○	○
Picnicking/grilling in the outdoors	○	○	○	○	○
Gardening	○	○	○	○	○
Orienteering	○	○	○	○	○
Hunting	○	○	○	○	○
Canoeing/rowing	○	○	○	○	○
Camping	○	○	○	○	○
Sea/lake swimming	○	○	○	○	○
Angling	○	○	○	○	○
Farming	○	○	○	○	○
Other activities in the outdoors	○	○	○	○	○

[If q28._14.resp.Response.Value.ContainsAny({_1,_2,_3,_4}) Then]

[q28A – STRING – single – Optional]
You indicated that you engage in other activities in the outdoors {#sq28A}. What other activities do you engage in?

[q29 – GRID – single – Must answer - Randomized]
Does the risk of getting tick bites and tick-borne diseases affect your engagement in any of the activities mentioned in the previous question? <i> Please provide one response per activity</i>

	Yes, to a very high degree	Yes, to a fairly high degree	Yes, to a low degree	No, not at all	Don't know
**Hiking/running in the outdoors**	◯	◯	◯	◯	◯
**Wild berry and mushroom picking**	◯	◯	◯	◯	◯
**Bicycling in the outdoors**	◯	◯	◯	◯	◯
**Horseback riding in the outdoors**	◯	◯	◯	◯	◯
**Picnicking/grilling in the outdoors**	◯	◯	◯	◯	◯
**Gardening**	◯	◯	◯	◯	◯
**Orienteering**	◯	◯	◯	◯	◯
**Hunting**	◯	◯	◯	◯	◯
**Canoeing/rowing**	◯	◯	◯	◯	◯
**Camping**	◯	◯	◯	◯	◯
**Sea/lake swimming**	◯	◯	◯	◯	◯
**Angling**	◯	◯	◯	◯	◯
**Farming**	◯	◯	◯	◯	◯
**Other activities in the outdoors**	◯	◯	◯	◯	◯

[If sTemp.resp.Response.Value.ContainsAny({_1,_2,_3}) Then bQ30 = True]

[If bQ30 = True Then]

[q30 – GRID – single – Must answer]
You have indicated that your leisure activities are affected by the risk of getting bit by a tick. How does this affect your quality of life?

	Very negatively<br>-5	-4	-3	-2	-1	Neither positively nor negatively<br>0	1	2	3	4	Very positively<br>+5

[q44 – STRING – single – Optional]
Would you like to share any further comments about ticks and tick-borne diseases or this survey/study?

<EndOfSurveyBM:>

Photo credits

The photographs in Questions 1 are used with the permission from Per Eikeseth Knudsen. Any further use of these photos must be cleared with the photographer (per.e.knudsen@uia.no).
